# Sexually selected dichromatism in the hihi *Notiomystis cincta*: multiple colours for multiple receivers

**DOI:** 10.1111/jeb.12417

**Published:** 2014-05-19

**Authors:** L K Walker, J G Ewen, P Brekke, R M Kilner

**Affiliations:** * Department of Zoology, University of CambridgeCambridge, UK; † Zoological Society of London, Institute of ZoologyLondon, UK

**Keywords:** Bateman gradient, plumage colour, selection gradient, sexual dichromatism, sexual selection

## Abstract

Why do some bird species show dramatic sexual dichromatism in their plumage? Sexual selection is the most common answer to this question. However, other competing explanations mean it is unwise to assume that all sexual dichromatism has evolved by this mechanism. Even if sexual selection is involved, further work is necessary to determine whether dichromatism results from competition amongst rival males, or by female choice for attractive traits, or both. Here, we test whether sexually dichromatic hihi (*Notiomystis cincta*) plumage is currently under sexual selection, with detailed behavioural and genetic analyses of a free-living island population. Bateman gradients measured for males and females reveal the potential for sexual selection, whilst selection gradients, relating reproductive success to specific colourful traits, show that there is stabilizing selection on white ear tuft length in males. By correlating colourful male plumage with different components of reproductive success, we show that properties of yellow plumage are most likely a product of male–male competition, whilst properties of the black and white plumage are an outcome of both male–male competition and female choice. Male plumage therefore potentially signals to multiple receivers (rival males and potential mates), and this may explain the multicoloured appearance of one of the most strikingly dichromatic species in New Zealand.

## Introduction

Sexual dichromatism, where one sex is brighter and more colourful than the other, is usually assumed to be the result of sexual selection (Kimball & Ligon, 1999). Thus, elevated reproductive success in more colourful males is assumed to be a consequence of more colourful individuals either having improved prospects in male–male contests (intrasexual selection) or having greater success at attracting females (intersexual selection). However, other explanations for sexual dichromatism have been proposed, such as predation pressure selecting for colour in the more conspicuous sex. Bright colouration may aid predator evasion, such as when the conspicuous zigzag bands of mate-searching male adders (*Vipera berus*) confuse visually hunting predators (Shine & Madsen, 1994), or may signal an individual's unprofitability as prey (Baker & Parker, 1979; Götmark, 1993; Götmark *et al*., 1997). Indeed, elaborate and colourful plumage does not always improve mating and reproductive success (Ligon & Zwartjes, 1995; Van Rooij & Griffith, 2012), suggesting that sexual selection need not necessarily be the default explanation for colourful plumage (Tarvin & Murphy, 2012).

How, then, can we establish that any sexual dichromatism is the consequence of sexual selection? One approach is to search directly for evidence of current sexual selection. Although sexual dichromatism may be the consequence of past selection pressures that are no longer operating, measuring the current patterns of selection is particularly relevant for the future evolutionary trajectory of plumage traits. The Bateman gradient (*β*_ss_), for example, quantifies the slope of the relationship between mating and reproductive success for each sex (Bateman, 1948; Arnold & Duvall, 1994). Sexual selection arises from competition for access to mates, and the Bateman gradient therefore gets to the heart of sexual selection by asking whether offspring production is constrained by mate availability. Bateman's third principle states that the slope of *β*_ss_ will be greater in the sex experiencing stronger selection, given that they have the most to gain from mating multiply (Arnold, 1994; Jones *et al*., 2002). Measuring Bateman gradients is therefore the first step in identifying the potential for sexual selection, without identifying specific traits that are under selection. If analysis of Bateman gradients indicates strong selection on mating success (i.e. sexual selection), then any plumage trait correlated with mating success will also be under sexual selection (Jones *et al*., 2005).

Unfortunately though, sexual selection is not the only possible explanation for a positive Bateman gradient, which may instead be the result of a number of alternative mechanisms (Gerlach *et al*., 2012). For example, a positive Bateman gradient may simply be a statistical artefact, given that the chance of detecting multiple mates increases when an individual has more offspring, or may be the result of fecundity selection, whereby the causal relationship is reversed and acquiring multiple mates is instead a consequence of having more offspring (Gerlach *et al*., 2012). The Bateman gradient, therefore, indicates whether sexual selection *might* be operating, although falls short of confirming that sexual selection definitely is operating (Klug *et al*., 2010; Gerlach *et al*., 2012). It also does not identify the specific phenotypic target of sexual selection. Despite these shortcomings, the Bateman gradient is widely acknowledged as having considerable utility in studies of sexual selection (Jones, 2009; Jennions & Kokko, 2010; Krakauer *et al*., 2011). And yet, there is a shortage of high-quality measures from the wild, particularly for females (Bergeron *et al*., 2012), largely due to the difficulties in genetically assigning parentage to all offspring in a wild population.

Another measure that quantifies selection in terms of total reproductive or mating success is the selection gradient (*β* and *γ*). This approach relates total reproductive/mating success to specific phenotypic traits. It can therefore potentially identify specific colourful traits that might be sexually selected, with the caveat that the plumage traits themselves might only be correlates of the true target of selection. Identifying which phenotypic traits contribute to variance in mating success is an important aim of sexual selection research (Gerlach *et al*., 2012) and can help pinpoint the target of selection suggested by a positive Bateman gradient. Indeed, there has been a recent request for studies of sexual selection to quantify multiple aspects of selection, rather than relying on single measures (Klug *et al*., 2010).

In many species, a male's route to acquiring reproductive success involves both intra- and intersexual selection. Amongst songbirds, for example, males commonly compete with rivals to acquire a territory (Part & Qvarnström, 1997; Marchetti, 1998; Andersson *et al*., 2002) and maintain exclusive mating access to a female (Delhey *et al*., 2003; Estep *et al*., 2005; Lehtonen *et al*., 2009; Eikenaar *et al*., 2011). Reproductive success may then be additionally enhanced by extra-pair fertilizations if males are perceived to be particularly attractive by other females (Double & Cockburn, 2000; Rubenstein, 2007; Chiver *et al*., 2008) and/or are able to outcompete the territorial male (Akcay *et al*., 2011). In some populations, some males fail to acquire a territory at all and rely exclusively on extra-pair fertilizations for any reproductive success (Sardell *et al*., 2010 and references therein). A male's ability to maximize reproductive success will therefore be a result of intra- and/or intersexual selection, and colourful traits associated with total reproductive success may be aligned to one or both of these mechanisms, depending on which component of reproductive success they enhance. Indeed, there is much value in evaluating the two mechanisms of sexual selection together, given that their interaction can have considerable bearing on the strength and form of sexual selection on a given trait (Hunt *et al*., 2009).

As no single type of analysis is sufficient to understand whether, and how, sexual selection is acting, we decided to use all the approaches outlined above to investigate the evolution of colourful male plumage in a sexually dimorphic and dichromatic passerine, the hihi (*Notiomystis cincta*). Surprisingly, the relevance of plumage colour for reproductive success in hihi is undetermined, despite it being one of the most strikingly dichromatic bird species in New Zealand. Male hihi weigh *c. *40 g and display colourful plumage, including carotenoid-based yellow shoulders (Ewen *et al*., 2006), melanin-based black heads and structurally produced white ear tufts. Conversely, females weigh *c. *32 g and have subtler, olive-brown plumage with a white wing bar. Hihi are largely socially monogamous, with a small proportion of socially polygynous males and high levels of extra-pair paternity (on average 68% of offspring within a brood are from extra-pair matings; Brekke *et al*., 2013). Males are either territorial and seek a combination of extra-pair fertilizations and within-pair fertilizations, or nonterritorial floaters who rely exclusively on extra-pair fertilizations. We began by testing the potential for sexual selection in this species by plotting Bateman gradients (*β*_ss_) for males and females. Next, we calculated selection gradients (*β* and *γ*) with respect to various properties of colourful male plumage. Finally, we established the importance of territoriality and within-pair and extra-pair success for achieving total reproductive success. We investigated the relationship between male plumage colour and these components of reproductive success by assessing which properties of colourful plumage were associated with (i) ability to acquire a territory, (ii) ability to withstand cuckoldry and (iii) success at gaining extra-pair fertilizations.

## Materials and methods

### Study species

Hihi are cavity nesters, and females lay up to two successful clutches per season with three to five eggs per clutch (Oliver, 1955). Females perform all nest building and incubation, and the majority of provisioning to nestlings (Ewen & Armstrong, 2000). Both males and females can, and do, reproduce in their first year (Low *et al*., 2007).

### Breeding season sampling

We studied a reintroduced hihi population on 220-ha Tiritiri Matangi Island (36°36′ S, 174°53′ E). This closed population has been intensely monitored since it was established in 1995 such that all breeding pairs and reproductive attempts are recorded (Ewen *et al*., 2011). In addition, biannual surveys are conducted using resighting of banded individuals to track individual survival accurately. Hence, the identity of breeding adults is well known.

Variation in properties of yellow plumage in this reintroduced population does not differ significantly from either the remnant (source) population (Hauturu Island: hue, *F*_6,18_ = 3.10, *P* = 0.17; chroma, *F*_6,18_ =0.95, *P* = 0.86; yellow brightness, *F*_6,18_ = 0.85, *P* = 0.72) or another reintroduced population (Kapiti Island: hue, *F*_7,18_ = 0.98, *P* = 0.90; chroma, *F*_7,18_ = 0.66, *P* = 0.44; yellow brightness, *F*_7,18_ = 0.65, *P* = 0.44; UV brightness, *F*_7,18_ = 1.60, *P* = 0.54). The only exception to this is for variation in UV brightness, which is significantly greater on Tiritiri Matangi than on Hauturu (*F*_6,18_ = 6.99, *P* = 0.02). We do not have equivalent data for the black and white plumage patches, and it is possible that patterns of selection may be different in populations with more or less variation in these traits.

This study focuses on the 2010/2011 austral breeding season. Based on the prebreeding season population survey, and observations of territoriality, nest building, incubation and provisioning behaviour, the identity of territorial males (*n* = 79), floater males (*n* = 17) and resident females (*n* = 98) was recorded. The greater number of resident females than territorial males reflects the fact that some territorial males were paired with multiple females. Upon completion of nest building, nests were monitored daily (except during incubation) to retrieve any unhatched eggs and dead nestlings. Tissue samples were collected from dead nestlings (*n* = 180; 81% of all dead nestlings) and from unhatched eggs that showed obvious signs of embryonic development (*n* = 60; 29% of all unhatched eggs). Tissue samples could not be collected from dead nestlings that were not found (*n* = 43), obviously, nor from unhatched eggs that showed no obvious signs of development (*n* = 149). Surviving nestlings were ringed at 21 days with a unique combination of one numbered metal ring and three colour rings, and a blood sample was taken by brachial venipuncture. Blood and tissue samples were stored in 95% ethanol and refrigerated for subsequent genotyping and paternity assignment. The identity of nestlings that survived to fledging (at about 30 days) was recorded (*n* = 243; 100% of all surviving nestlings sampled). Of a possible 675 eggs laid, a total of 483 offspring (unhatched embryos + dead nestlings + surviving nestlings) were sampled.

### Genetic analysis and parentage assignment

Genomic DNA was extracted from tissue and whole blood using the ammonium acetate precipitation method (Nicholls *et al*., 2000). All individuals were genotyped at a set of 19 autosomal selectively neutral microsatellite loci (see detailed methods in Brekke *et al*., 2009, 2013).

The parentage of each sampled offspring was assigned using a maximum-likelihood method in the program colony 2.0 (Wang & Santure, 2009). This program allows estimation of parentage under a promiscuous mating system and incorporates full and half-sibship relationships to increase the statistical power (Wang, 2004). colony 2.0 provides a posterior probability value for each maternal and paternal assignment, which usually increases when behavioural information on potential parentage is incorporated. We included maternal information from behavioural observations, and there was a high congruence between social and genetic maternity assignment (99%), confirming the power of this method. Candidate fathers (*n* = 91 males) were those males known to be alive and for which genotypes were available (95% were genotyped). The probability of the true parents being in the candidate lists was set at 0.90 for both fathers and mothers. Only parentage assigned with 95% confidence was accepted for use in subsequent analyses. Paternity was confidently assigned to 82% of offspring.

### Plumage colour measurement

In October 2010, at the commencement of the 2010–2011 breeding season, males were caught in mist-nets or feeding station traps for plumage colour measurement. Reflectance spectra were recorded using a USB-2000 spectrometer (Ocean Optics Inc., Dunedin, FL, USA), a DT-Mini Lamp (Deuterium Tungsten Halogen source) and a reflectance probe, following methods described in Walker *et al*. (2013). Repeated measurements were taken from the left and right yellow shoulder patches (3 repeats on each side, 6 in total), the black head (3 repeats in total) and the left and right white ear tufts (3 repeats on each side, 6 in total) in all males caught (*n* = 89 males). The reflectance probe was lifted and replaced between repeat measurements within a plumage region. Repeatability measures did not assess degree of symmetry (in the case of yellow and white plumage patches, where measurements were taken from left and right sides), but rather the repeatability across all measurements made within yellow, black and white plumage regions. Repeated measurements of yellow patch size (using digital photographs and a scale rule, and subsequent area calculation in imagej) and white ear tuft length (using digital callipers) were also made, according to Walker *et al*. (2013). Repeatability of these measures is reported below.

### Plumage colour analysis

To accommodate the fundamental differences between avian and human photoreception (Kelber *et al*., 2003; Osorio & Vorobyev, 2005, 2008; Cuthill, 2006), reflectance spectra were analysed using models in tetrahedral colour space (Endler & Mielke, 2005; Stevens *et al*., 2009; Stoddard & Prum, 2011). Hue, saturation and luminance variables for each colour patch were extracted according to methods described in Walker *et al*. (2013). In brief, photon catch values for the single and double cones were calculated using ‘d65’ irradiance spectra and blue tit (*Cyanistes caeruleus*) spectral sensitivities (a species with an ultraviolet-shifted shortwave-sensitive cone type; Hart *et al*., 2000). Our measure of luminance, which describes the perceived lightness of a patch, was the double-cone photon catch values. The standardized single-cone catch data for each individual were plotted in avian tetrahedral colour space (Endler & Mielke, 2005), and saturation, the amount of colour compared to white light, was calculated as the distance from the centre of the colour space. We calculated hue, the colour type (e.g. blue versus red), by performing a principal component analysis (PCA) on a covariance matrix of the standardized single-cone data (see Walker *et al*., 2013 for full details).

One-way anovas were used to assess repeatability of different plumage colour variables (following Lessells & Boag, 1987). All plumage colour variables demonstrated significantly higher between-individual than within-individual variation (*P* < 0.0001). Repeatability was relatively high in most cases, with *R* values > 0.94 for patch size measurements (yellow area and white length) and *R* values ranging from 0.38 to 0.39 for yellow descriptors (hue, saturation and luminance), 0.25 to 0.31 for black descriptors and 0.48 to 0.60 for white descriptors. These values are consistent with other published repeatability values for colourful traits (Saino *et al*., 1999; Biard *et al*., 2005; Budden & Dickinson, 2009), justifying their use. Repeated measures per individual were averaged for use in subsequent analyses. Yellow hue and saturation were highly correlated (*r* = 0.94, *P* < 0.001), and we chose to use saturation rather than hue as it most consistently reflects feather carotenoid content across species (Saks *et al*., 2003; McGraw & Gregory, 2004). Hue and saturation were also correlated in black (*r* = −0.22, *P* = 0.04) and white (*r* = 0.48, *P* = 0.004) plumage, and we again used saturation rather than hue. Luminance is encoded independently of colour and is analysed separately by visual systems, justifying its separate analysis for yellow, black and white patches.

### Statistical analysis

#### Bateman gradients (*β*_ss_)

A nonparametric mood test was used to test for a difference in variance between male and female mating success (number of mates) and between male and female reproductive success (both number of fertilizations and number of offspring fledged). A mood test is a rank-based test that compares the variances of two samples that are not normally distributed (Conover, 1971), as is the case for male and female mating and reproductive success. Number of mates was identified as the number of different partners an individual had fertilizations with. Bateman gradients (*β*_ss_) were calculated by performing a linear regression of reproductive success (number of fertilizations or number of offspring fledged) on mating success (number of mates; Arnold & Duvall, 1994). The number of mates, sex and an interaction between number of mates and sex were included as explanatory variables. Ninety-one males and 91 females were included in these analyses.

#### Linear selection gradients (*β*)

Linear (directional) selection gradients (*β*) were calculated as the coefficients from a multiple linear regression of relative male fitness on standardized male traits (Lande & Arnold, 1983). Three separate measures of male fitness were used – number of mates, number of fertilizations and number of offspring fledged – and all were transformed to relative male fitness by dividing by the population mean. The male traits considered were yellow saturation, yellow luminance, yellow patch size, black saturation, black luminance, white saturation, white luminance, white ear tuft length and tarsus length, and all were standardized (mean = 0, variance = 1) for selection analyses. Age was not included because this is not a trait on which selection can act. However, first-year males had significantly lower values than older males for some traits (yellow saturation, yellow area, black saturation, white saturation, white luminance and white length), which could obscure any within-age relationships for these traits. For this reason, we standardized these traits within age classes (first-year and older males) and then combined these two age classes for selection analyses (Sheldon & Ellegren, 1999). All males of known age with complete colour measurements, and that were included as candidate fathers in the parentage assignment (i.e. had potential to be assigned as fathers), were included in these analyses (*n* = 79).

Residuals were not normally distributed, and although this assumption is not necessary for estimating selection gradients (Lande & Arnold, 1983), it is necessary for significance testing. Splitting selection analysis into parameter estimation and significance testing is commonly implemented (Mitchell-Olds & Shaw, 1987; Fairbairn & Preziosi, 1996; Sheldon & Ellegren, 1999), and we therefore used generalized linear models (GLMs) with Poisson errors (response = count) to test the significance of the regression coefficients. Standardized multivariate selection analysis was devised to estimate selective pressures after accounting for correlations between phenotypic traits (Lande & Arnold, 1983). However, severe multicollinearity will affect the results, primarily by elevating standard errors and making it harder to reject the null hypothesis (Mitchell-Olds & Shaw, 1987). We therefore assessed the extent of multicollinearity in our models by computing the variance inflation factor (VIF) for each trait. A VIF is the factor by which the standardized unexplained variance is inflated as a result of intercorrelation between explanatory variables (Sokal & Rohlf, 1995), such that a large VIF indicates that the explanatory variables are highly correlated. Generally, a VIF > 10 indicates harmful multicollinearity (Kennedy, 1992). The VIF for a given explanatory variable *i* is calculated as 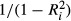 where 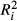 is the *R*^2^ from a regression of *i* against all other explanatory variables. The VIFs for our explanatory variables ranged from 1.11 to 1.49, indicating that any correlations between the male traits were not large enough to cause concern.

#### Decomposing contributions to reproductive success

We decomposed the relative contribution of the different elements of male reproductive behaviour (territory ownership, mate guarding and extra-pair mating) to reproductive success. For all males, the importance of territoriality for total reproductive success was investigated by fitting GLMs with Poisson errors and a log link function. The response (total reproductive success) was either total number of fertilizations or total number of offspring fledged. For territorial males only, the importance of within-pair versus extra-pair success for total reproductive success was determined by partitioning the variance in total reproductive success into its component parts (Webster *et al*., 1995). As total reproductive success (T) is the sum of within-pair (W) and extra-pair (E) reproductive success, the variance in total reproductive success (var(T)) is the sum of variance in within-pair success (var(W)), variance in extra-pair success (var(E)) and twice the covariance between the two (cov(W, E)), that is, var(T) = var(W) + var(E) + 2 cov(W, E) (Webster *et al*., 1995). Dividing by the mean of total reproductive success squared will standardize these variances. The standardized variance in total reproductive success is known as the opportunity for sexual selection (*I*) and is a measure of the maximum possible strength of selection. Reproductive success was measured as number of fertilizations and as number of offspring fledged.

#### Plumage colour and components of reproductive success

Next, we investigated the importance of plumage colour for components of reproductive success by asking the following questions: (i) Are males with certain plumage properties more likely to hold a territory? (ii) Having acquired a territory, are males with certain plumage properties less likely to be cuckolded? (iii) Are males with certain plumage properties more successful at gaining extra-pair and/or within-pair fertilizations? To answer questions (i) and (ii), we fitted GLMs with binomial errors and a logit link function. In the territory holding model, the binary response variable was whether or not a male was a territory holder (*n* = 83 males of known age and with complete colour measurements). In the cuckoldry model, the two-vector response was composed of the number of fertilizations on a territory that were the territorial male's and the number that were another male's (*n* = 65 territorial males; only territorial males can be cuckolded). To answer question (iii), we fitted GLMs with Poisson errors and a log link function, where the response was a count of either the number of extra-pair fertilizations (*n* = 79 territorial and floater males; all males eligible for extra-pair fertilizations) or number of within-pair fertilizations (*n* = 65 territorial males; only territorial males eligible for within-pair fertilizations). The explanatory variables were as above (in ‘selection gradients’), in addition to age and age^2^ being included to control for age. The age^2^ term was included because hihi have previously shown a quadratic relationship between age and reproductive performance (Low *et al*., 2007). A negative age^2^ term will indicate that intermediate-aged males have the highest reproductive success, whilst a positive age^2^ term indicates that old and young males have the greatest success.

Because the inclusion of the age terms introduced high levels of multicollinearity (age VIF = 22.77 − 25.86 across the three models), we took a multimodel inference approach using the package MuMIn in the program r (Grueber *et al*., 2011). This allowed us to generate a candidate set of models that considered all possible combinations of explanatory variables whilst excluding correlated variables. Models were ranked by AICc value, and model-averaged coefficients were generated by natural averaging over models with *Δ*AICc < 2 (Grueber *et al*., 2011). All explanatory variables were standardized (mean = 0, variance = 1), which is necessary for model averaging (Grueber *et al*., 2011). Because there was overdispersion in the territory defence model, and in the extra-pair and within-pair models, we specified the dispersion parameters from quasibinomial and quasi-Poisson models (2.61, 2.92 and 2.33, respectively).

#### Nonlinear selection gradients (*γ*)

For those traits identified as correlates of components of reproductive success (see section ‘Plumage colour and components of reproductive success’ above), we also calculated nonlinear selection gradients (*γ*). We could not do this for all traits because of limited sample size and therefore chose to focus on traits that results from section ‘Plumage colour and components of reproductive success’ above suggested could be under some form of selection. Nonlinear selection gradients were calculated by including squared terms for the traits of interest in regression models [as detailed in section ‘Linear selection gradients (*β*)’ above]. Stabilizing selection (where intermediate values of a trait have higher relative fitness) would be indicated by a significant negative coefficient value. Disruptive selection (where extreme values of a trait have higher relative fitness) would be indicated by a significant positive coefficient value.

## Results

### Bateman gradients (*β*_ss_)

**Figure 1:**
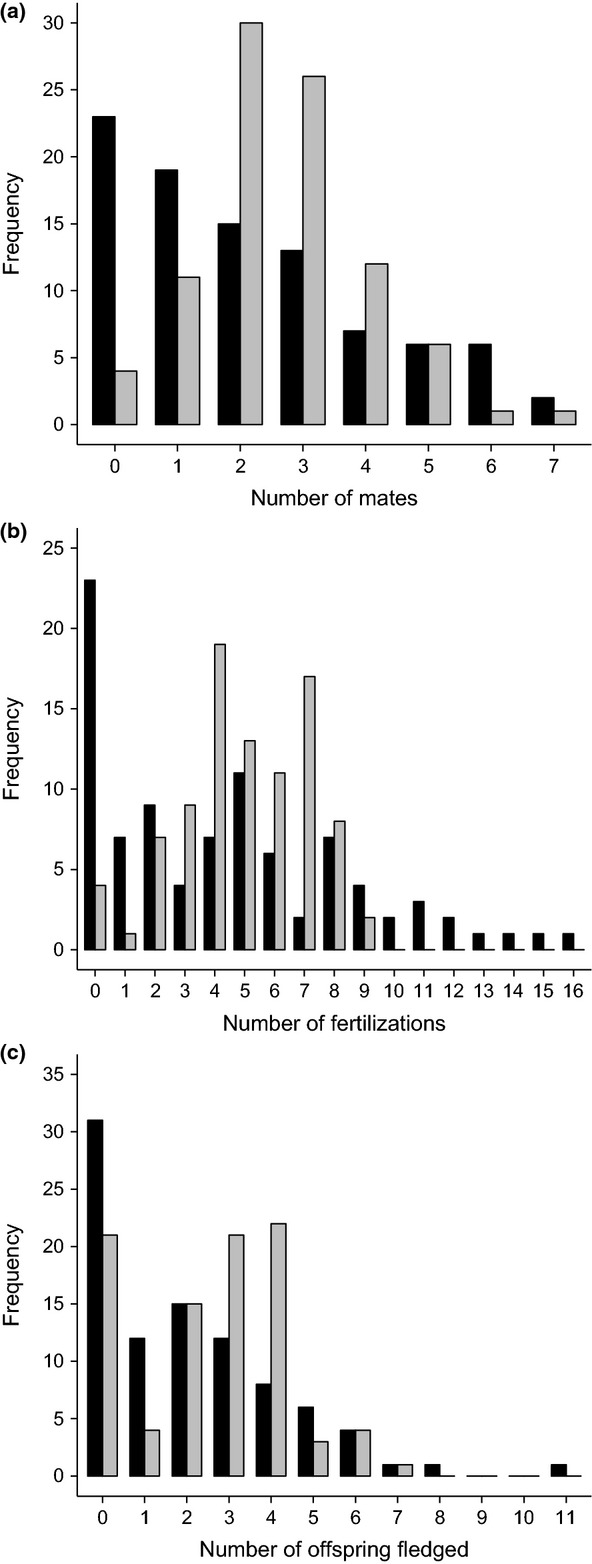
Frequency histograms of (a) mating success, (b) fertilization success and (c) fledged offspring success for males (black bars) and females (grey bars). Males showed greater variance than females in mating success and fertilization success, but variance was equal for number of offspring fledged.

**Figure 2:**
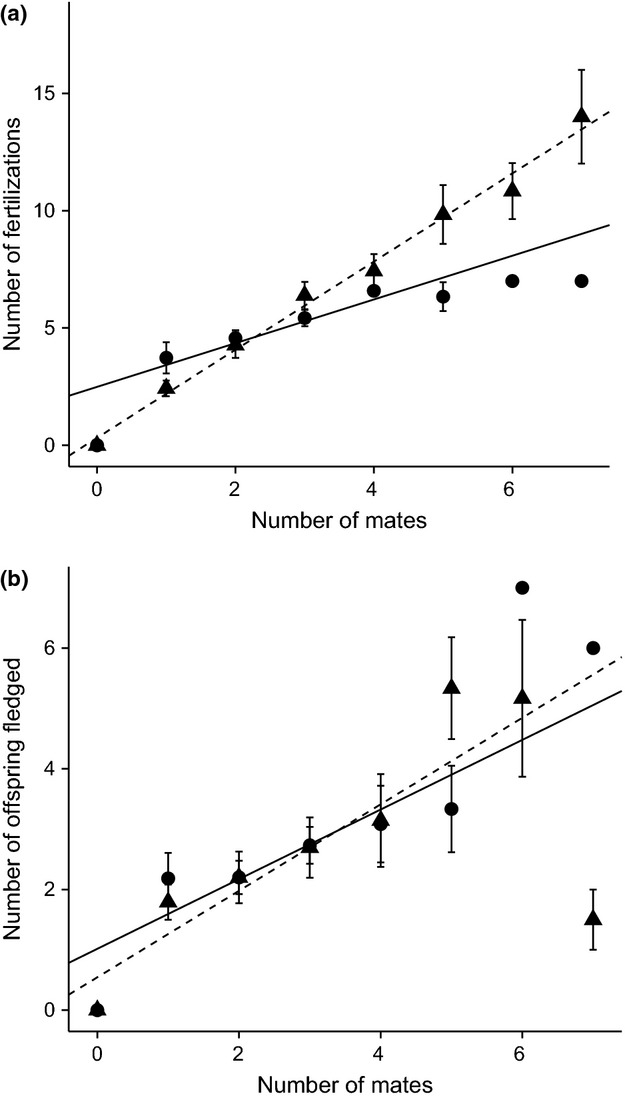
Bateman plots of reproductive success against number of mates for males (triangles and dashed lines) and females (circles and solid lines). Reproductive success measured as (a) number of fertilizations and (b) number of offspring successfully fledged. Points are means ± SE, and lines are predicted from linear regressions: (a) *y* = 1.88*x* + 0.32 (male), *y* = 0.93*x* + 2.49 (female); (b) *y* = 0.72*x* + 0.54 (male), *y* = 0.58*x* + 1.02 (female).

Variance in the number of mates was significantly greater for males than for females (*z*_1,90_ = 5.19, *P* < 0.001; Fig.1a), as too was variance in the number of fertilizations (*z*_1,90_ = 5.75, *P* < 0.0001; Fig.1b). Variance in the number of offspring that successfully fledged, however, did not differ between the sexes (*z*_1,90_ = 1.33, *P* = 0.18; Fig.1c). Reproductive success increased with increasing number of mates for both males and females (Fig.2). This was the case when reproductive success was measured as the number of fertilizations and when it was measured as the number of offspring fledged (Fig.2; Table1). The rate of increase was greater for males than for females in the number of fertilization model, but in the number of offspring fledged model, the rate of increase was equal for males and females (Fig.2, Table1). Results were qualitatively the same when reproductive success and mating success were relativized by dividing by their respective means.

**Table 1:** Bateman gradients (*β*_ss_) for males and females (*n* = 182 birds). Reproductive success is measured as (a) number of fertilizations or (b) number of offspring fledged. The significant interaction between number of mates and sex in (a) indicates that the rate of increase in number of fertilizations with increasing number of mates was greater for males than for females. Significant terms highlighted in bold.

	Estimate ± SE	*t*-Value	*P*-value
(a) Number of fertilizations			
Intercept	2.49 ± 0.42	5.98	<0.0001
Number of mates	**0.93 ± 0.14**	**6.61**	**<0.0001**
Sex*	**−2.17 ± 0.50**	**−4.34**	**<0.0001**
Number of mates: sex	**0.95 ± 0.17**	**5.57**	**<0.0001**
(b) Number of offspring fledged			
Intercept	1.02 ± 0.39	2.61	0.01
Number of mates	**0.58 ± 0.13**	**4.37**	**<0.0001**
Sex*	−0.47 ± 0.47	−1.01	0.31
Number of mates: sex	0.14 ± 0.16	0.88	0.38

* Estimate relative to female.

### Linear selection gradients (*β*)

**Figure 3:**
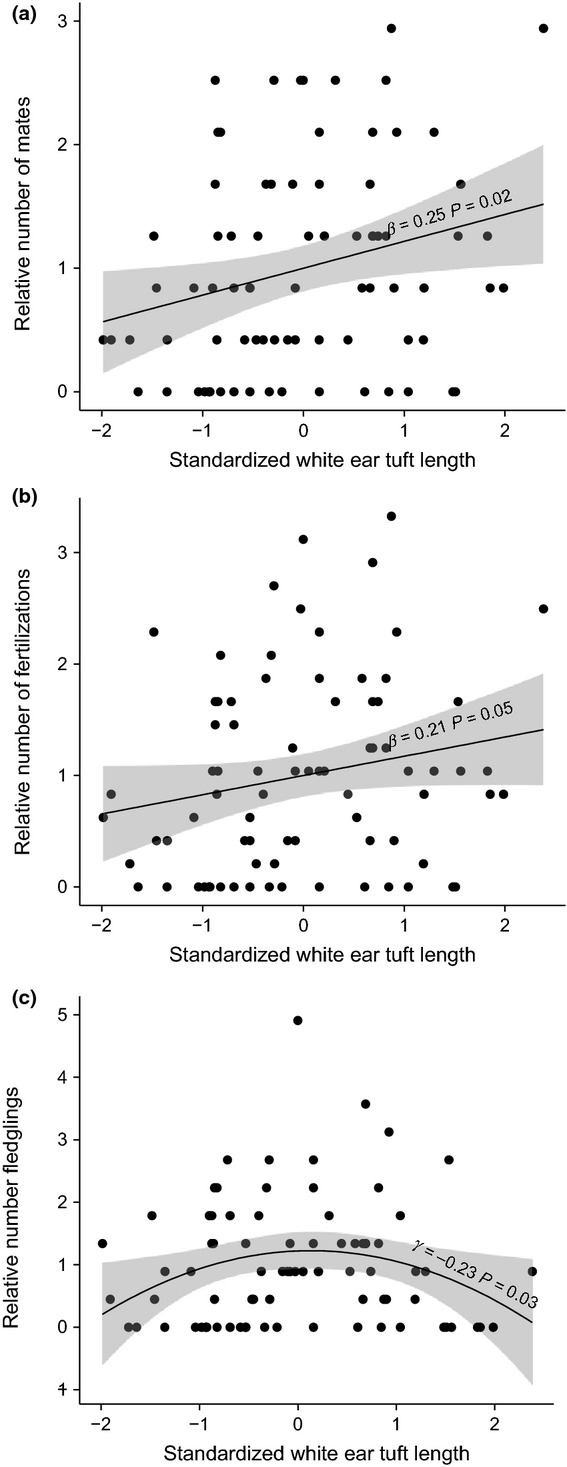
Directional and stabilizing selection on white ear tuft length: (a) directional selection on white ear tuft length via relative number of mates, (b) directional selection on white ear tuft length via relative number of fertilizations and (c) stabilizing selection on white ear tuft length via relative number of offspring fledged. Regression lines are plotted with shaded grey area showing 95% confidence interval around the regression line.

There was significant positive directional selection on white ear tuft length, both when fitness was measured as relative number of mates (*β *= 0.25 ± 0.10; Table2; Fig.3a) and when it was measured as relative number of fertilizations (*β *= 0.21 ± 0.10; Table2; Fig.3b). There was, however, no evidence for directional selection on ear tuft length when fitness was measured as relative number of offspring fledged (*β *= 0.04 ± 0.13; Table2). There was also no evidence for directional selection on any of the other plumage traits considered, nor on body size (Table2).

**Table 2:** Standardized linear selection gradients (*β*) for male plumage traits and body size, where male fitness is measured as relative number of mates, relative number of fertilizations and relative number of offspring fledged (*n* = 79 males). Significant results highlighted in bold.

Male trait	Relative number of mates			Relative number of fertilizations			Relative number fledglings		
	*β *± SE*	*t*-Value†	*P*-value†	*β *± SE*	*t*-Value†	*P*-value†	*β *± SE*	*t*-Value†	*P*-value†
Yellow saturation	−0.080 ± 0.116	−0.59	0.56	−0.061 ± 0.121	−0.40	0.69	−0.059 ± 0.146	−0.37	0.72
Yellow luminance	−0.018 ± 0.102	−0.12	0.90	−0.105 ± 0.106	−0.94	0.35	−0.136 ± 0.128	−1.06	0.30
Yellow area	−0.031 ± 0.103	−0.33	0.74	0.017 ± 0.107	0.10	0.92	0.018 ± 0.129	0.13	0.90
Black saturation	0.043 ± 0.107	0.28	0.78	0.043 ± 0.111	0.30	0.77	0.071 ± 0.134	0.49	0.63
Black luminance	0.027 ± 0.100	0.23	0.82	0.017 ± 0.104	0.14	0.89	0.026 ± 0.125	0.21	0.83
White saturation	0.087 ± 0.102	0.67	0.51	0.065 ± 0.106	0.49	0.62	0.051 ± 0.128	0.32	0.75
White luminance	−0.020 ± 0.100	−0.08	0.94	−0.042 ± 0.104	−0.30	0.77	−0.032 ± 0.125	−0.22	0.82
White length	**0.250 ± 0.100**	**2.46**	**0.02**	**0.212 ± 0.104**	**1.97**	**0.05**	0.041 ± 0.125	0.28	0.78
Tarsus length	0.124 ± 0.102	1.25	0.22	0.125 ± 0.106	1.19	0.24	0.024 ± 0.127	0.18	0.86

* Parameter estimation from linear regression.

† Significance testing from generalized linear model.

### Decomposing contributions to reproductive success

Being territorial was important for maximizing total reproductive success. Territorial males had significantly greater fertilization success (*z*_1,77_ = 6.48; *P* < 0.001), and fledged significantly more offspring (*z*_1,77_ = 4.42, *P* < 0.001), than nonterritorial floater males. Partitioning total variance in reproductive success into its component parts revealed that, for territorial males, variance in extra-pair success was the greatest contributor to male reproductive success (Table3). Variance in within-pair success explained a smaller percentage of total variance, and covariance a smaller percentage still (Table3). This was the case both when reproductive success was measured as number of fertilizations and when it was measured as number of offspring fledged (Table3). The negative covariance between within-pair and extra-pair success, in both cases, suggests that there is a trade-off between the two, although this is very weak (Table3).

**Table 3:** Variance in total reproductive success (var(T)) partitioned into within-pair variance (var(W)), extra-pair variance (var(E)) and within-pair extra-pair covariance (cov(W, E)). Reproductive success is measured either as number of fertilizations or as number of offspring fledged. Standardized value is absolute value divided by mean total reproductive success squared.

	Number of fertilizations			Number of offspring fledged		
Absolute value	Standardized value	% Total variance	Absolute value	Standardized value	% Total variance	
var(T)	16.22	0.50	100	5.08	0.72	100
var(W)	6.54	0.20	40.36	2.00	0.29	39.45
var(E)	9.76	0.30	60.21	3.12	0.45	61.50
2 cov(W, E)	−0.09	−0.003	−0.57	−0.05	−0.007	−0.95

### Plumage colour and components of reproductive success

Males with a larger yellow patch size were significantly more likely to hold a territory than males with a smaller yellow patch size (Table4a). Intermediate-aged males were also more likely to be territorial, as evidenced by a significant negative age^2^ term (Table4a). No other plumage properties influenced the likelihood of a male holding a territory, and neither did tarsus length (Table4a).

**Table 4:** Results of generalized linear models, following model averaging, investigating whether plumage colour predicts a male's ability to (a) acquire a territory (*n* = 83 males), (b) avoid cuckoldry (*n* = 65 territories), (c) gain extra-pair fertilizations (*n* = 79 territorial and floater males) and (d) gain within-pair fertilizations (*n* = 65 territorial males). Models (a) and (b) have binomial errors, and estimates are in logits. Models (c) and (d) have Poisson errors, and estimates are in logs. All estimates have been standardized. Only terms that appear in model averaged set are listed. Significant terms highlighted in bold.

	Estimate ± SE	*z*-Value	*P*-value	Relative importance
(a) Territory acquisition				
Intercept	2.94 ± 0.63	4.610	<0.0001	
Yellow area	**1.36 ± 0.44**	**3.05**	**0.002**	**1.00**
Age^2^	−**0.67 ± 0.24**	**2.70**	**0.007**	**1.00**
White luminance	0.59 ± 0.37	1.57	0.12	0.69
White saturation	0.61 ± 0.49	1.23	0.22	0.42
Black luminance	−0.26 ± 0.33	0.77	0.44	0.21
Yellow luminance	0.35 ± 0.38	0.90	0.37	0.16
Black saturation	0.21 ± 0.39	0.54	0.59	0.06
(b) Avoiding cuckoldry				
Intercept	−0.15 ± 0.16	0.88	0.38	
Yellow luminance	−**0.39 ± 0.18**	**2.14**	**0.03**	**1.00**
Tarsus length	0.16 ± 0.17	0.92	0.35	0.54
White saturation	0.09 ± 0.15	0.59	0.56	0.17
White luminance	−0.10 ± 0.18	0.54	0.59	0.16
Black saturation	0.07 ± 0.15	0.47	0.64	0.14
Age^2^	−0.04 ± 0.12	0.35	0.73	0.07
Black luminance	−0.08 ± 0.22	0.37	0.72	0.07
Yellow area	0.09 ± 0.17	0.52	0.60	0.07
(c) Extra-pair fertilizations				
Intercept	0.99 ± 0.25	4.00	<0.0001	
Black luminance	**0.29 ± 0.12**	**2.33**	**0.02**	**1.00**
Age^2^	−0.35 ± 0.26	1.36	0.18	1.00
White length	**0.48 ± 0.13**	**3.61**	**0.0003**	**0.56**
Yellow luminance	−0.13 ± 0.13	0.97	0.33	0.56
Age	**0.69 ± 0.20**	**3.44**	**0.0006**	**0.44**
White saturation	0.08 ± 0.12	0.68	0.49	0.30
Tarsus length	0.07 ± 0.14	0.52	0.60	0.20
(d) Within-pair fertilizations				
Intercept	1.09 ± 0.11	9.56	<0.0001	
Yellow luminance	−0.19 ± 0.12	1.54	0.12	1.00
White luminance	−0.11 ± 0.12	0.93	0.35	0.40
Tarsus length	0.12 ± 0.13	0.89	0.37	0.40
Black saturation	0.09 ± 0.10	0.88	0.38	0.40
White saturation	0.08 ± 0.10	0.79	0.43	0.26
Yellow area	0.08 ± 0.13	0.58	0.56	0.04

Having acquired a territory, males with lighter yellow plumage (i.e. higher luminance) were more likely to be cuckolded than males with darker yellow plumage (Table4b). No other terms considered, including age, tarsus length and other colour traits, influenced a male's likelihood of being cuckolded (Table4b).

Finally, males with lighter black plumage (i.e. higher luminance) and longer white ear tufts had greater extra-pair fertilization success than males with darker black plumage and shorter white ear tufts, respectively (Table4c). Also, older males had greater extra-pair fertilization success than did younger males (Table4c). No other plumage traits explained whether a male was likely to be successful at getting extra-pair fertilization success, and neither did tarsus length (Table4c). None of the variables considered predicted a territorial male's within-pair fertilization success (Table4d).

### Nonlinear selection gradients (*γ*)

There was significant stabilizing selection on white ear tuft length when fitness was measured as relative number of offspring fledged (*γ* = −0.23 ± 0.10; Table5; Fig.3c). There was no stabilizing or disruptive selection on white ear tuft length when fitness was measured as either relative number of mates or relative number of fertilizations (Table5). There was also no evidence of either stabilizing or disruptive selection on yellow area, black luminance or yellow luminance (Table5).

**Table 5:** Standardized nonlinear selection gradients (*γ*) for yellow area, black luminance, yellow luminance and white length, where male fitness is measured as relative number of mates, relative number of fertilizations and relative number of offspring fledged (*n* = 79 males). Significant result highlighted in bold.

Male trait	Relative number of mates			Relative number of fertilizations			Relative number of fledglings		
	*γ* ± SE*	*t*-Value†	*P*-value†	*γ* ± SE*	*t*-Value†	*P*-value†	*γ* ± SE*	*t*-Value†	*P*-value†
Yellow luminance	−0.054 ± 0.072	−0.75	0.45	−0.077 ± 0.074	−1.09	0.28	−0.069 ± 0.085	−0.96	0.34
Yellow area	−0.030 ± 0.079	−0.39	0.70	0.013 ± 0.082	0.22	0.82	0.093 ± 0.094	1.20	0.23
Black luminance	−0.052 ± 0.043	−1.16	0.25	−0.048 ± 0.045	−1.09	0.28	−0.059 ± 0.051	−1.11	0.27
White length	−0.082 ± 0.087	−1.18	0.24	−0.093 ± 0.090	−1.19	0.24	−**0.233 ± 0.103**	−**2.27**	**0.03**

* Parameter estimation from linear regression.

† Significance testing from generalized linear model.

### Unknown fertilizations

We were not able to assign paternity to every single fertilized egg in the population for two reasons. Firstly, approximately 71% of unhatched eggs (149 of 209) did not show visible signs of development, and any developing embryo that may have been present could not be sampled. Secondly, the bodies of approximately 19% of chicks that hatched but died before fledging (43 of 223) were not recovered. As a consequence, approximately 28% of the eggs laid (192 of 675) were not sampled and could not have paternity assigned (although approximately 12% of nondeveloping eggs were likely infertile; Hemmings *et al*., 2012). If the probability of embryo/chick death is biased according to father phenotype, then these unknown fertilizations could bias our results (e.g. if embryos/chicks that die are more likely to be sired by males with short-ear tufts, then the fertilization success of short-ear tuft males will be underestimated). We therefore tried to establish whether the male traits that we found to be important for mating/reproductive success (ear tuft length, black luminance, yellow luminance) relate to embryo and nestling mortality, because we were able to assign paternity to some dead offspring.

The fathers of offspring that died (both as embryos and as chicks) and the fathers of offspring that survived did not differ in either black luminance (*t*_1,263_ = −0.39, *P* = 0.70) or yellow luminance (*t*_1,263_ = −0.18, *P* = 0.85). This suggests that the importance of black luminance for determining extra-pair fertilization success, and the importance of yellow luminance for cuckoldry, should be unaltered if unknown paternity fertilizations (all from dead offspring) were included in the analyses. The fathers of offspring that died had significantly longer white ear tufts than the fathers of offspring that survived (*t*_1,263_ = −2.04, *P* = 0.04). This result is interesting and might explain why there was directional selection via mating and fertilization success, but stabilizing selection via fledgling success.

### Note on multiple testing

A large number of statistical tests have been performed in this study, which will inflate the chance of making type I errors and falsely rejecting the null hypothesis. We have not performed any correction for multiple comparisons on the grounds that such results will be no less prone to error (Perneger, 1998). Instead, we caution that results should be interpreted with the understanding that multiple statistical tests have been performed.

## Discussion

**Figure 4:**
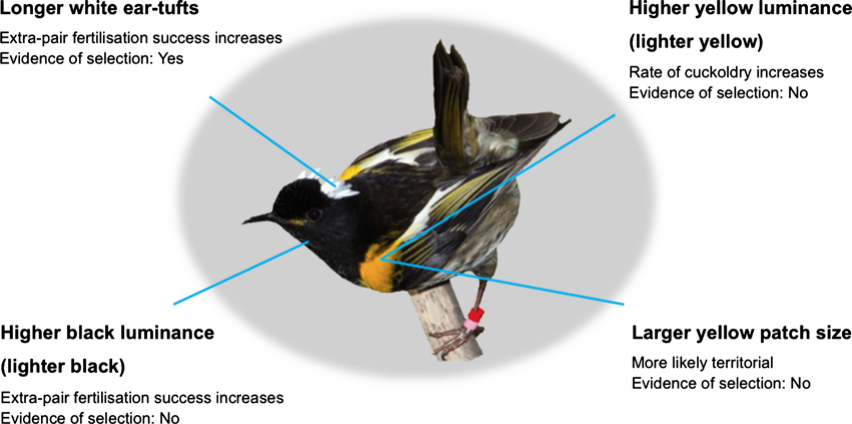
A male hihi with distinctive carotenoid-based yellow, melanin-based black and structurally produced white plumage. Text indicates how components of reproductive success change with increasing expression of the plumage trait, and whether there was any evidence for selection on these traits (photograph: Brent Stevenson).

Both male and female hihi exhibited positive Bateman gradients, indicating that there is potential for sexual selection to be operating in both sexes. Measuring selection gradients in relation to male plumage traits revealed that there was positive directional selection for white ear tuft length via mating and fertilization success, but stabilizing selection for white ear tuft length via fledgling success. In addition, we found that different plumage traits were associated with different components of male reproductive success: a larger yellow plumage patch increased the likelihood of being territorial, lighter yellow plumage (higher luminance) increased the likelihood of being cuckolded, and lighter black plumage (higher luminance) and longer white ear tufts increased extra-pair fertilization success (Fig.4). However, because extra-pair reproductive success was the greatest contributor to total male reproductive success, it was only white ear tuft length, the plumage trait most strongly associated with extra-pair success, which showed evidence of being under selection (Fig.4).

What do these results tell us about the role of male–male competition and female choice in the evolution of these plumage traits? Territoriality is usually considered to be primarily an outcome of competition amongst males to acquire and defend a territory (Part & Qvarnström, 1997; Marchetti, 1998; Andersson *et al*., 2002). Yellow plumage in hihi appears to be a consequence of male–male competition, because it is associated with both territory acquisition and mate defence (avoiding cuckoldry). For example, the 15 males who failed to acquire a territory had significantly smaller yellow plumage patches than the 68 territorial males.

In contrast, extra-pair fertilizations, which often contribute substantial variance to male reproductive success (Vedder *et al*., 2011), are usually assumed to be under female control (Jennions & Petrie, 2000). Female superb fairy-wrens (*Malurus cyaneus*), for example, undertake predawn forays into other territories for the express purpose of seeking out extra-pair mates (Double & Cockburn, 2000). However, in some species, females appear to have limited control over extra-pair paternity. If extra-pair copulations occur on her own territory, for example, a female's choice of extra-pair mate will be restricted to only those males able to evade her social mate, meaning that the role of male–male competition will be more prominent (Akcay *et al*., 2011). Furthermore, forced extra-pair copulations occur in some species, with males aggressively coercing females into mating with them (Clutton-Brock & Parker, 1995). Sexual coercion has been suggested as a third mechanism of sexual selection, and there may be strong selection on traits that improve a male's chances of successfully forcing copulations (Clutton-Brock & Parker, 1995). Aggressive extra-pair copulations are common in waterfowl (Adler, 2010) and have also been reported in some passerines (Westneat & Stewart, 2003), including hihi (Castro *et al*., 1996; Ewen *et al*., 2004; Low, 2005).

In hihi, both solicited and forced extra-pair copulations occur, and both are believed to result in fertilizations (Brekke *et al*., 2013). Given the high frequency of forced extra-pair copulation in hihi (83% of all EPCs observed by Low, 2005), the traditional view that extra-pair fertilizations, and therefore traits associated with extra-pair fertilization success, are driven primarily by female choice may not apply. Nevertheless, solicited extra-pair copulations do occur (Low, 2005), and perhaps at a greater rate than estimated if they are less conspicuous than the highly visible and vocal forced extra-pair copulations. Furthermore, it is possible that female hihi exert some additional control via post-copulatory female choice, as seen, for example, in feral fowl where females eject the sperm of subdominant males that have coerced them into mating (Pizzari & Birkhead, 2000). Indeed, the positive female Bateman gradient that we present here suggests that females do stand to gain from mating multiply and would therefore benefit from seeking out extra-pair males. Thus, in contrast to traits associated with territoriality, there may be some scope for black luminance and white ear tuft length, the traits associated with extra-pair fertilization success, to have evolved by both inter- and intrasexual selection, and potentially also by sexual coercion. Perhaps, the black plumage serves to provide a contrasting backdrop for the white ear tufts during their display. Indeed, behavioural observations confirm that white ear tufts are used in displays towards other males, during contests at feeding sites and on territories, and towards females, during courtship and copulation (L. K. Walker & J. G. Ewen, pers. obs.). In the context of sexual coercion, perhaps a plumage trait would be selected if it is correlated with a physical trait that enhances a male's ability to force copulations. Indeed, it may even pay females to attend to male signals of coercive ability, if the costs of resistance outweigh the costs of immediate submission to a coercive male.

We have demonstrated that male hihi with longer white ear tufts have greater mating and fertilization success (Fig.3a, b), but that their offspring are more likely to die before fledging than males with intermediate ear tufts (Fig.3c). We interpret this pattern as evidence of stabilizing selection on ear tuft length, although we cannot explain why it exists. Stabilizing selection is seen when honest signals incur costs (Hinde *et al*., 2010). Does this mean male ear tufts are costly signals (Grafen, 1990; Zahavi & Zahavi, 1997)? The answer is no, because the costs associated with longer ear tufts are not borne directly by the signaller (the male) but by his offspring (through reduced viability) and, indirectly, by the female. We therefore find no evidence from this study consistent with lengthy white ear tufts in hihi bearing associated costs for males. However, this does not mean that white ear tufts are not costly in some other way, and further work from other populations and across multiple years would be required to test this possibility more comprehensively.

Despite yellow and black plumage being correlated with territoriality and extra-pair success, respectively, we did not find evidence that these traits were under either linear or nonlinear selection (Fig.4). One possibility is that we had insufficient power to detect selection. However, our sample sizes do fall within the range of those reported in equivalent studies that were generally capable of detecting various forms of selection (Sheldon & Ellegren, 1999; McGlothlin *et al*., 2005; Westneat, 2006). Another consideration is that perhaps with a different combination of ecological factors, as might be generated, for example, in different years, these plumage traits might become the target of sexual selection. Indeed, even a narrow range of environmental variation can affect the expression of male traits and their attractiveness to females (Ingleby *et al*., 2014). Thus, the persistence of multiple components in the hihi's sexually dichromatic plumage could simply reflect the spectrum of ecological conditions experienced by the population over several years and, consequently, result from fluctuating selection on a battery of different plumage types (Brooks & Couldridge, 1999). Extrapolating more generally, we might then expect to see more complex sexual ornamentation, involving multiple component parts, in highly variable environments (where the environment includes the sensory system of the potential mate (Brooks, 2002) as well as wider ecological circumstances). One example of this is in the lark bunting (*Calamospiza melanocorys*), where frequent changes in female mate preference, driven by a highly variable social and/or ecological environment, result in fluctuating sexual selection on a large number of ornamental male traits (Chain & Lyon, 2008).

Ecological conditions, and their consequences for evolutionary potential, will also be influenced by management interventions. Despite being a wild population, the hihi on Tiritiri Matangi Island do receive a level of management that has some potential to reduce the intensity of sexual selection on plumage traits. Providing artificial nest boxes and supplementary food may have generated a scenario in which poorer-quality individuals are able to gain territories and/or reproductive success when they might otherwise (i.e. in unmanaged habitats) have been unable to do so. This would reduce the degree of variation in reproductive and/or mating success, by increasing the success of individuals that previously had limited success, and therefore reduce the potential for sexual selection. To test this idea would require conducting an equivalent study on a hihi population that receives no management (currently only the Hauturu Island population), which, almost by definition, would be a considerable (but worthwhile) challenge.

A final note of caution is that all of the approaches used here involve correlational analyses and so do not identify cause and effect (Grafen, 1987): we cannot tell whether trait expression enhances reproductive success or whether reproductive success enhances trait expression. It is not inconceivable, for example, that reproductive activity could influence the luminance of black plumage. Frequent extra-pair forays, which involve aggressive encounters with other birds and tussles in the abrasive leaf litter (Castro *et al*., 1996), might cause males to undergo greater feather wear, reducing the melanin content of black feathers and thus making them appear brighter (McGraw *et al*., 2005). Likewise with selection gradients, although we measured a number of traits, we cannot exclude the possibility that white ear tuft length is simply correlated with an unmeasured trait that is the true target of selection (Krakauer *et al*., 2011).

In conclusion, we provide a rare quantification of male and female Bateman gradients in a natural population. We have shown that the multiple colourful plumage traits of male hihi are relevant for multiple receivers. Yellow plumage displays appear to be directed primarily towards other males in contests over territories. Black and white plumage displays are seemingly directed both towards other males, during disputes for extra-pair fertilizations, and towards females either to charm them or to force extra-pair success. We show evidence of stabilizing selection on white ear tuft length, during this year of study, but recognize that different ecological pressures during other years may increase the importance of yellow and black traits such that they also come under selection.
